# Effects of Zinc Supplementation on Nutritional Status in Children with Chronic Kidney Disease: A Randomized Trial

**DOI:** 10.3390/nu11112671

**Published:** 2019-11-05

**Authors:** Marlene Fabiola Escobedo-Monge, Guido Ayala-Macedo, Graciela Sakihara, Silvia Peralta, Ana Almaraz-Gómez, Enrique Barrado, J. M. Marugán-Miguelsanz

**Affiliations:** 1 Faculty of Medicine, National University of San Marcos, Cangallo 818, 15001 Cercado de Lima, Peru; 2 National Institute of Child Health, Paediatric Nephrology Service, Avenida Brasil 600, 15083 Breña, Peru; chela_sakihara@yahoo.com; 3 Faculty of Medicine, Valladolid University, Avenida Ramón y Cajal, 7, 47005 Valladolid, Spain; 4 Faculty of Food Science and Technology, National Agrarian University, Avenida la Molina, s/n, 15024 La Molina, Lima, Peru; silviacesi@yahoo.com; 5 Department of Public Health and Preventive Medicine, Faculty of Medicine, Valladolid University, 47005 Valladolid, Spain; aalmaraz@med.uva.es; 6 Department of Analytical Chemistry, Science Faculty, University of Valladolid, Campus Miguel Delibes, Calle Paseo de Belén, 7, 47011 Valladolid, Spain; ebarrado@qa.uva.es; 7 Department of Paediatrics of the Faculty of Medicine, Valladolid University, Section of Gastroenterology and Pediatric Nutrition, University Clinical Hospital of Valladolid, 47005 Valladolid, Spain; jmmarugan@telefonica.net

**Keywords:** serum zinc concentration, hypozincemia, chronic kidney disease, serum albumin, C-reactive protein, underweight, undernutrition, stunted growth

## Abstract

Background: Zinc is an essential micronutrient for human beings and its deficiency affects their normal growth and development. Objective: The main aim was to evaluate the effect of two doses of zinc supplementation (ZS) on the nutritional status in chronic kidney disease (CKD) children. Methods: A randomized-trial multicentric study was conducted in 48 CKD (23 females) patients under 18-years-old, for a year. At random, participants took 30 or 15 mg/day of ZS, respectively. Anthropometric measurements and biochemical analysis were performed. Hypozincemia was determined by serum zinc concentration (SZC) using atomic absorption spectrophotometry. The positive or negative change in patients’ body mass index (BMI) Z-score, serum albumin, zinc and C-reactive protein (CRP) levels were used to evaluate the effect of ZS. Results: Mean SZC was normal before and after ZS. Despite ZS, there were no significant changes in serum albumin, zinc and CRP levels. A positive and significant association was observed between SZC and serum albumin before (*p* = 0.000) and after (*p* = 0.007) ZS. In both groups of ZS, there was a small but positive and significant change in body mass and normalization in BMI Z-score, hypoalbuminemia, hypozincemia and high CRP, especially with 30 mg/day of ZS. Conclusions: Zinc supplementation may be beneficial for nutritional status in children and adolescents with CKD.

## 1. Introduction

Over the last 10 years [1], chronic kidney disease (CKD) also known as chronic renal failure (CRF) is increasingly recognized as a global public health concern and an important contributor to morbidity and mortality [2]. While the burden of CKD is reasonably well defined in developed countries, increasing evidence indicates that the CKD burden may be even greater in developing countries [3]. Globally, the incidence and prevalence of CKD adults is increasing rapidly due to the rapid increase in the prevalence of risk factors such as diabetes, hypertension and obesity [2], which, in the future, will also produce a greater burden of CKD [1]. 

Even though it is more common in adults than in children, CKD is a chronic disease with severe long-term consequences. Moreover, although there are similarities between adult and pediatric populations with CKD, there are unique features and issues in childhood not evident in adults, such as the impact of the disease on growth [4]. The severe growth retardation is extremely common among CKD children and occurs in up to 35% of this population before the end-stage renal disease (ESRD) takes place [5]. Since the nutritional point of view, adequate nutrition and periodic evaluation are key components to prevent the development of protein-energy malnutrition (PEM) and meet the patient’s vitamin and mineral needs [6].

The most important objective in CKD children is that they have a normal childhood. Nevertheless, this can be difficult because CKD creates a complex pathologic environment characterized by metabolic alterations that affect nutrient intake, metabolism and energy expenditure, predisposing patients to the development of malnutrition and an increased risk of morbidity and mortality [7]. The general decrease in nutritional intake, dietary restrictions, poor intestinal absorption, inflammatory state, metabolic acidosis and dialysate losses all put the CKD patient at risk of micronutrient deficiencies, which may contribute to comorbidities such as anemia, cardiovascular disease, and metabolic imbalances [8]. Furthermore, in CKD children malnutrition, metabolic acidosis, mineral and bone disorders, anemia and fluid and electrolyte abnormalities are risk factors that contribute to impaired growth [9].

Zinc is one of the most important [10] and essential trace elements required by all living organisms for many physiologic functions [11], with three major biological roles catalytic, structural and regulatory ones [10]. It is the second most abundant metal in mammalian tissues, after iron [12], with almost 90% of that found in muscle and bone [13]. Likewise, the cellular Zn^2+^ concentrations are nearly as high as those of major metabolites like the ATP are [14]. Zinc is an essential cofactor that influences the expression and activity of numerous enzymes, transcription factors and regulatory proteins [15]. It is indispensable for the structure and function of at least 3000 proteins [14] and cellular components and plays an important role in human physiology from its involvement in the proper function of the immune system to its role in cellular growth, cell proliferation, cell apoptosis, as well as in the activity of numerous zinc-binding proteins [10]. Additionally, zinc is an antioxidant with anti-inflammatory properties, and regulates innate and adaptive immune responses, which makes it crucial for resistance to infection [16].

The recent recognition of fundamental regulatory functions of Zn^2+^ ions released from cells or within cells; links this nutritionally essential metal ion to numerous human diseases. Zinc and its role in organ pathophysiology as well as in genetic, metabolic, chronic and infectious diseases are covered [14]. A high level of zinc has also been found in other organs including the brain, heart, kidney, liver, prostate, pancreas, lung, skin and gastrointestinal (GI) tract. Maintaining a constant state of cellular zinc nutrition is essential for normal function. In the periphery, zinc homeostasis is a highly regulated and coordinated process that involves uptake through intestinal epithelial cells and reabsorption via the kidneys; changes in the absorption/excretion of zinc in the GI tract are the primary mechanisms for maintaining zinc homeostasis in the body [17,18].

Zinc is a multifunctional metal compatible with satisfactory growth, health and well-being [10]. It is known to regulate growth, neuronal development and immunity [19], and its deficit can affect the development of multiple organs, including the brain, lungs, skeleton, kidneys and heart [20]. This micronutrient plays a critical role in the microtubules function since microtubule formation involves tubulin polymerization, a process that is decreased by zinc deficiency (ZnD) [21]. ZnD is comorbid with CKD and worsens kidney complications. Besides, oxidative stress is implicated in the detrimental effects of ZnD [22]. NADPH oxidases (Nox) are the primary enzymes that contribute to renal reactive oxygen generation. Experimental findings show that ZnD exacerbates diabetic kidney damage by enhanced oxidative damage, fibrosis, and renal dysfunction [15].

Even though the needs for zinc in CKD patients have not been established [7], the National Kidney Foundation Kidney Disease Outcomes Quality Initiatives (KDOQI) suggests that both children and adults should receive the dietary reference intakes (DRI) for zinc. KDOQI recommends that intake for infants (4.0–5.0 mg/day) and children (5.0–9.5 mg) to be monitored every 4–6 months and supplements be given if necessary [6]. These doses may not be enough for CKD children in developing countries. At that point, we need to highlight four important aspects. First, ZnD is little known in children with CKD. Second, clinical studies have reported that patients treated by conservative treatment (CT), long-term hemodialysis (HD) [23], or continuous ambulatory peritoneal dialysis (CAPD) exhibit ZnD. Third, the predominant effect of renal insufficiency on zinc homeostasis is hypozincemia due to increased urinary zinc excretion and ZnD may also play a role in the progression of renal failure. Finally, ZS studies showed attenuation slowing down the progression of kidney disease [24]. Therefore, the main aim of this study was to assess the effect of two doses of ZS on nutritional status in CKD children.

## 2. Materials and Methods

A multicenter, simple-blind (partially masked) essay on the effect of two doses of ZS was conducted in Lima, Peru. The study took place in tertiary health care institutions, at the Pediatrics Nephrology Service of the National Institute of Child Health (NICH) and the Social Security of Health (EsSalud)—Edgardo Rebagliati Martins Hospital and Metropolitan Hemodialysis Center (MHC), reference centers for CKD across the country. The study was performed following the Declaration of Helsinki, and the Ethics Committee of NICH and EsSalud approved the trial protocol. The ‘Consejo Nacional de Ciencia, Tecnología e Innovación Tecnológica’ (CONCYTEC-349-06-96-OAI), provided the financial support after the corresponding approval by the local ethics committee, and the corresponding follow-up of this study. The persons legally responsible for participants gave their informed consent for inclusion before participating in this study.

**Figure 1:**
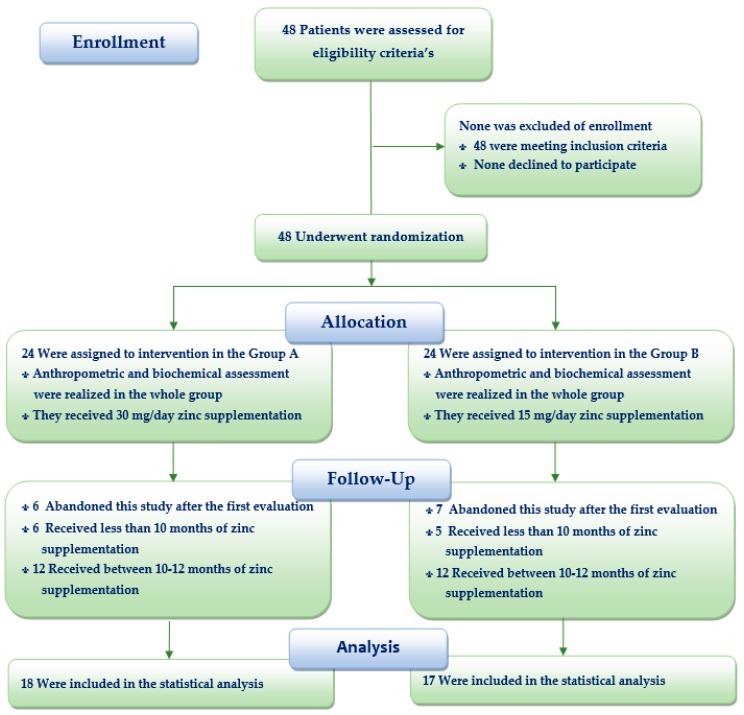
Flow diagram the effect of two doses of zinc supplementation on nutritional status in children with chronic kidney disease.

A sample size adjusted to 15% losses of 41 patients was obtained to compare the two doses of ZS, taking into account that about 130 children with CKD per year receive treatment in nephrology services of Lima and Callao. The proportions were compared using the type of unilateral test, with a 95% confidence level, 90% statistical power and 15% accuracy. Subjects were taken consecutively from nephrology services. However, because some children lived in Lima but came from provinces, it was decided to increase the number of patients to 48 adjusted to 27% losses during the first few months of this study, following the same protocol. Participants were eligible for the study if they were 1–18 years of age and with CKD regardless of the stage of their disease. They continued with their medical, nutritional and physical activity protocolized treatment corresponding to their nutritional and CKD status provided by their nephrology service, which was not modified by researchers. Patients neither were supplemented with zinc nor had recombinant human growth hormone (rhGH) therapy throughout the trial. Acute infection, hospitalization and refusal to participate were exclusion criteria (Figure 1).

This randomized trial was designed in two phases. The diagnosis of baseline nutritional status was made in the first phase of two months, a washing period before participating in this study. In the second experimental phase of 12 months, children were randomly assigned following simple randomization procedures (a random number table) to the first or second parallel groups, in a 1:1 ratio, to receive one of two possible doses of oral ZS. Participants received either 30 mg/day (equivalent to 6.8 mg elemental zinc; group A) or 15 mg/day (equivalent to 3.4 mg elemental zinc; group B). This last group was not a placebo group but a group of patients treated with 15 mg/day of zinc, a daily dose of zinc requirement commonly recommended to increase bone density [23]. The available information supports the premise that children with CKD are a population at risk of ZnD. Therefore, it was decided to supplement both groups and not to use a control group, investigating which of the doses used in this study would have the greatest effect on their nutritional status.

The same researcher carried out the sequence generation, allocation concealment as well as the implementation step. Physicians allocated to the groups knew the ZS assigned, the outcome assessors and the data analysts were kept blinded to the assignment. In each center, a bottle with the oral dose of zinc (tablets) was provided for the first month after phase one, and again every month after the anthropometric follow-up evaluations during their participation. It was recommended to participants to take the single daily oral dose of zinc during or after a meal without any other medication and after dialysis in HD and CAPD to avoid its elimination during dialysate. Simultaneously, each patient took their corresponding replacement therapy of vitamins and minerals according to the stage of their disease.

Anthropometric assessments of weight (W), height (H), mid-arm circumference (MAC) and triceps skinfold thickness (TSF) using standard techniques were performed on enrolment and every follow-up month. The equipment was calibrated daily, and the anthropometric team was standardized monthly; the technical errors of measurement for length (0.45 ± 0.05 cm) and weight (40 ± 5 g) were considered acceptable. The measure and Z-score of weight-for-age (W/A), height-for-age (H/A), weight-for-height (W/H), nutritional index (NI), body mass index (BMI), BMI-height-age, mid-arm muscle area (MAMA), fat-free mass (FFM) and fat mass (FM) were calculated using Frisancho [25] and Orbegozo Tables [26]. The growth rate (GR) was calculated based on the height information in their medical records. The cut-off to value anthropometric parameters was <−2 standard deviation score (SDS).

Fasting blood samples at the start and the end of the experimental study were collected (before dialysis in patients in HD). Blood was collected in sterile disposable plastic syringes, which were previously washed with acid to eliminate any contamination. Each sample (3 mL) was centrifuged at 3000 rpm for 5 min. The samples were brought to the laboratory of the ‘Instituto de Investigación Nutricional’ in Lima, reference center endorsed by the World Health Organization (WHO), using an atomic absorption spectrophotometer. Even though SZC between 70 and 120 μg/dL was considered normal [27], SZC below 70 μg/dL in boys and girls under 10 years, and below 70 and 74 μg/dL in women and men aged 10 years or more, respectively, were used as cut-off points to evaluate hypozincemia [28]. The activity of C-reactive protein as an inflammatory marker (CRP > 4 U/L) and serum albumin as visceral protein reserve (≤3.5 g/dL) were examined by standardized methods.

Nutritional assessment by anthropometric and biochemical data was used to determine whether a CKD patient was well-nourished or malnourished (comorbidities). The primary endpoint on the effect of ZS on nutritional status was to achieve at least a 10% difference between the two groups from baseline to 12 weeks in BMI, W/A and H/A Z-score, serum zinc, albumin and CRP levels. The variation from ≥−2 SDS towards normalization (between <−2 SDS to <2 SDS) in BMI, W/A and H/A Z-score was considered a positive change and the change towards ≥−2 SDS or 2 SDS reflected a negative variation. The improvement from hypozincemia, hypoalbuminemia and high CRP status towards their normalization was considered a positive modification, and from their normal levels toward their serum albumin and ZnD and increase CRP’s level meant a negative change. The secondary endpoint was the significant change (increase or decrease in the measurement) in patients’ anthropometric and biochemical indicators from initial presentation to 12th month after the onset of treatment.

The intention-to-treat analysis was the strategy of pre-specified analysis in the protocol of this prospective study. Statistical Data Management was implemented with SPSS/PC software (IBM Corp., Armonk, NY, USA). The distribution of anthropometric (quantitatively and their corresponding Z-scores) and biochemical evaluation data, was described as mean ± SDS and range. The comorbidities and causes of CKD in this series were expressed as percentages. The units of analysis for comparisons between intervention groups were the 12-month child-period of observation. Differences between treatment groups and between comorbidities were analyzed by Student’s *t*-tests and a McNemar test, respectively. Wilcoxon rank test was used to compare the values from the baseline and 12 months after ZS. Pearson’s correlation coefficients were performed to evaluate significant associations between variables. The simple and multiple linear regression analysis was calculated to study the relationships between two and more correlations. The analysis of variance (ANOVA) was used to look for interactions in analytical values between sexes, age groups and treatment. The modification (positive and negative) in anthropometric and biochemical evaluations was classified and analyzed. The proportion of patients with positive or negative change was compared between treatment groups by X^2^ test with Yates’s correction and Fisher’s exact test. The significance level was set at *p* < 0.05.

## 3. Results

A total of 48 patients (23 females) were screened for eligibility, enrolled and included in two treatment groups. The proportion and characteristics of the participants at the start did not differ between the study groups (Table 1). The mean age was 12.8 ± 4 years with a median of 14 and range from 1 to 18 years (23 children and 25 adolescents). There were 69% of patients in HD (33/48 cases), 10% in CAPD (5/48 cases) and 21% in CT (10/48 cases). The 60.4% of the cases came from the NICH, and 39.6% from the EsSalud. The most common causes of CKD in this series are presented in Table 2.

The duration of ZS was 9.3 ± 3.9 months with a median of 11 months and the range was 1–12 months. In both treatments groups, 50% received less than 10 months of ZS and another 50% between 10–12 months of ZS. The participants contributed 324 12-month child-periods of observation (73%) and follow-up anthropometric assessment over the whole study. The 27% (13/48) did not receive the planned intervention of ZS and abandoned the study after the first anthropometric and biochemical assessment (six of the group A and seven of the group B). The reasons for non-adherence to the treatment were that one of participants was transplanted and other 12 patients were in dialysis (three cases) and CT (nine cases) had personal reasons for not following these treatments. Two patients in HD, who were transplanted after the first 3 months of ZS, did not complete this study. For this reason, the authors decided to analyze all the outcomes on condition that these patients completed at least one month of ZS. Therefore, from this moment in time of the study, the modified intention-to-treat analysis was the analysis strategy, which was performed.

During the 12-month child-periods of observation, caregivers reported no adverse events such as nausea, vomiting, loss of appetite, abdominal cramps, diarrhea and headache, fever and lethargy after taking ZS [28] or anything associated with an acute infection. At the start of the study, the prevalence of stunting (83.3% low H/A) and underweight (77.1% low W/A) were higher than undernutrition (10.4% low BMI). There was one case with wasting (low W/H) and another case with obesity (BMI > 2 SDS) before and after ZS. In the whole series, there was a small but significant difference in W/A, H/A, W/H, MAC, FM and W/A and H/A Z-score (Table 3).

The mean SZC was normal at the start (75 ± 15.5 μg/dL, CI 95% 69.8–80.2 μg/L) and the end (73.5 ± 17.4 μg/dL, CI 95% 66.2–80.82 μg/L) of this study. Participants in HD were the only ones who completed two blood samples. Males had a higher mean of SZC (75.6 and 75 μg/dL) than females (74.6 and 72 μg/dL). Although the mean SZC after ZS was slightly lower than at the beginning, there was no significant difference in group A (from 78 to 71.9 μg/dL) nor group B (from 77.9 to 75.4 μg/dL). The mean serum albumin was normal before and after ZS. Even though CRP decreased after ZS, this change was not significant. There was no significant difference by sex or treatment groups in serum albumin or CRP levels.

**Figure 2:**
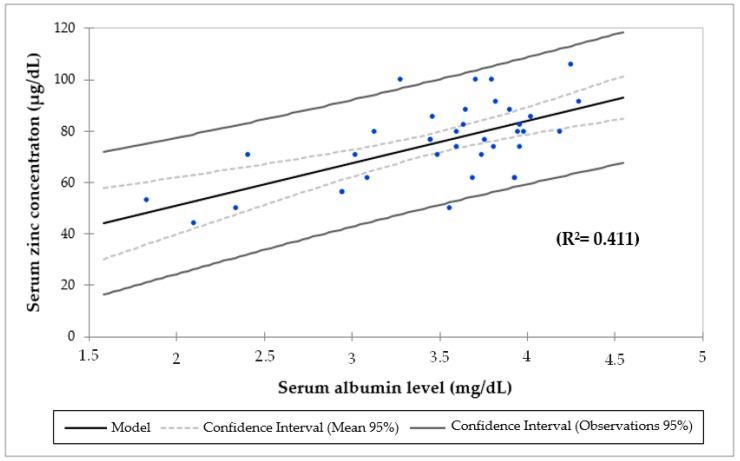
Linear regression analysis of serum zinc and albumin concentration before zinc supplementation.

**Figure 3:**
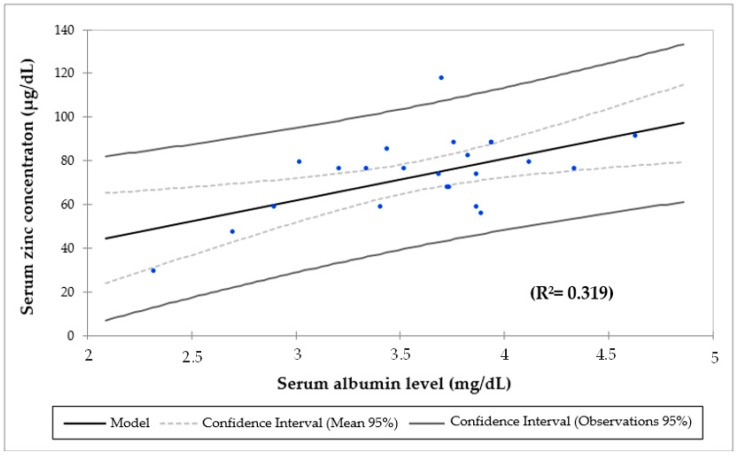
Linear regression analysis of serum zinc and albumin concentration after zinc supplementation.

There was a positive and significant association between SZC and serum albumin before (*r* = 0.64; *p* = 0.000) and after ZS (*r* = 0.55; *p* = 0.007). Linear regression analysis demonstrated that SZC was correlated with serum albumin in all participants before (*r* = 0.41, *p* = 0.000; Figure 2) and after ZS (*r* = 0.32, *p* = 0.007; Figure 3). Systemic inflammation was common at baseline and the end of the study. Even though there was an increase in patients with elevated CRP (from 40% to 54.5%), with hypoalbuminemia (from 36.8% to 37.5%) and with hypozincemia (from 40.5% to 41.7%), these changes were not significant. In the beginning, there were three patients with hypozincemia, hypoalbuminemia and a high CRP. In the end, these same patients had hypozincemia, and two of them had hypoalbuminemia and a high CRP as well.

**Figure 4:**
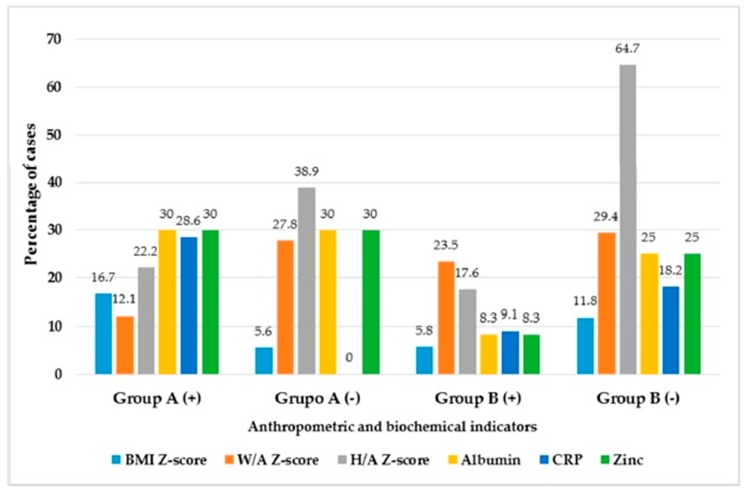
Percentage of cases with positive (+) or negative (−) change by groups of zinc supplementation. BMI: body mass index, W/A: weight-for-age, H/A: height-for-age, CRP: C-reactive protein, Albumin; serum albumin level, Zinc: serum zinc concentration.

Regarding the secondary endpoint, in the whole series after 12 months of ZS, a dose-effect was observed as a small but positive and significant change in their anthropometric assessments (Table 4), especially in group A (Table 5). In the multiple regression analysis of W/A, H/A, W/H, MAC, TSF, FFM and FM, by groups after ZS, R Square indicated that BMI can be explained mainly by W/A (*p* = 0.017), H/A (*p* = 0.033) and FFM (*p* = 0.035) in the group A, and by W/A (*p* = 0.006) in the group B. The provision of the two treatments caused no significant changes in the serum albumin, zinc and CRP levels, as well as hypozincemia, hypoalbuminemia and CRP cases. In connection with the primary endpoint, there were more patients with positive change in BMI Z-score (*p* = 0.020), serum albumin (*p* = 0.032), CRP levels (*p* < 0.0001) and SZC (*p* = 0,032) in group A compared with group B. There was more than 10% of the expected difference between groups. In contrast, there were more patients with a negative change in H/A Z-score and CRP levels (*p* < 0.050) in group B and serum albumin (*p* < 0.050) in group A (Table 6 and Figure 4).

## 4. Discussion

CKD is a major health problem worldwide with increasing incidence and prevalence that is threatening to bring about the onset of a real ‘epidemic’ [29]. However, there are no reliable statistics about the prevalence of CKD in most of the developing world [3,30]. While data are available regarding vitamins in CKD, little is known about the trace elements, especially in children [31]. Even though dietary zinc deficiency affects 20%–25% of the world’s population [32] especially adolescents and postmenopausal women [33], zinc deficiency is rarely seen as a serious deficit [34]. However, data from the WHO [35] reported that zinc deficiency is the fifth largest health risk factor in developing countries and the eleventh in the world [36]. The main prevalence of ZnD is observed in developing countries in Africa, Asia and Central America as well as in Andean countries [37]. 

In Lima and Callao, the total number of children under 18-years-old in HD (58 cases) and CAPD (81 cases) was 139 patients. The prevalence of children under 18 years of age who were receiving renal replacement therapy (RRT) was 14 children per million populations (pmp). In 2015, the main aetiologies were primary glomerulopathies, chronic interstitial nephropathies and congenital aetiology [38], information that contrasts with our results (Table 2). Regardless of CKD aetiology, is accompanied by ZnD [39], which contributes to kidney damage [15,22]. In HD patients, zinc-deficient status is associated with immune system disturbances, poor nutritional status, atherosclerosis and high rates of hospitalization due to infections [16]. Conversely, improved zinc status is associated with alleviating oxidative stress, inflammation, dyslipidemia and malnutrition in dialysis patients [40]. Therefore, the main objective of this study was to assess the effect of two doses of ZS on the nutritional status in children with CKD.

In CKD children, since adequate nutritional status is important for normal growth and development, a careful monitoring of nutritional status is essential [6]. The Chronic Kidney Disease in Children study revealed that 7%–20% of pediatric CKD patients had protein-energy wasting (PEW) [41]. Depending on the clinical parameters used to define malnutrition, a prevalence of 20%–45% has been reported in children with CKD in various studies [42]. Malnutrition has been shown to increase the risk of morbidity and mortality in both adult and pediatric patients with CKD [43]. In this study, despite ZS, there was a slight increase in underweight (from 77.1% to 80%) and undernutrition cases (from 10.4% to 22.9%). However, there was one single girl, who improved her obesity status (BMI from 3.5 to 2.5 SDS) after 30 mg/day for 3 months. The role of zinc dyshomeostasis in obesity was also confirmed by the results of supplementation trials. In particular, the administration of 30 mg/day zinc gluconate for 1 month resulted in a significant decrease in body weight and BMI values as well as serum triglycerides (TG) concentrations [44].

Additionally, there was another 10-year-old girl with chronic malnutrition and osteodystrophy, and CKD due to chronic interstitial nephritis, who at the beginning of the study could not walk alone and needed help to move around her house, hospital and other places. After 11 months of 30 mg/day of ZS, she improved her W/H, BMI and CRP. The most important improvement was that she could walk without help at the end of the study [45]. ZS may be the reason why this girl improved her health situation, because ZnD causes a marked reduction in circulating GH and IGF-I concentrations [46], and the administration of exogenous GH or IGF-I does not correct zinc deficiency-associated growth defects [47]. The zinc concentration is relatively high in bone, cartilage and teeth [48]. In addition to zinc’s active role in collagen formation in the epitheses, zinc ions are promoters of bone remodeling by osteoblast proliferation [49], and they contribute to extracellular matrix calcification through the synthesis of matrix proteins in osteoblasts [50].

Well-known complications of childhood CKD are a significant delay in growth and short stature [51]. Mean H/A Z-score below the lower limit of normal has been reported in most studies [52]. Even though, there was a slight decrease in cases with low H/A Z-score from 83.3% to 82.9% after ZS, this percentage continues to be high. Furthermore, 65.7% with H/A Z-score >2.5 SDS is important because short stature is associated with increased morbidity and mortality. This situation is worrying because Wong et al. (2000) reported a 14% increase in death risk for each SDS decrease in height, in children with ESRD [53]. Moreover, poor growth has serious consequences, including hospitalization, mortality and poor quality of life [54]. Part of these issues may be due to an increased risk of infection in malnourished patients [51,52]. 

In spite of the nutritional status of this series, results show that the mean SZC before (75 ± 15.5 μg/dL, *p* = 0.005) and after ZS (73.5 ± 17.4 μg/dL, *p* = 0.016) were normal and differed significantly from the National Health and Nutrition Examination Survey (NHANES) 2011–2014 study (82.7 ± 0.6 μg/dL) performed in 4347 participants [55]. Nevertheless, after the 12-month supplementation with two doses of zinc, there were no significant changes in the mean SZC of the children. On the contrary, El-Shazly et al. (2015) studied this subject in 40 children between 5 and 18 years old on regular HD (mean age 13.8 ± 3.1 years), after 90 days of a daily ZS (50–100 mg zinc sulphate (equivalent to 11–22 mg elemental zinc)), according to age, sex and nutritional status of each child. They found that the mean SZC had significantly increased from 53.2 ± 8.15 µg/dL to 90.75 ± 12.2 µg/dL (*p* = 0.001) in comparison with the control group [56]. A recent randomized study by Tonelli et al. (2015) showed that low dose supplementation fails to correct low zinc status in the HD population [57]. In this study, doses less than 50 mg/day of ZS may be the reason why in the whole series and by groups SZC was not corrected.

Furthermore, Tonelli et al. demonstrated in 2009, that zinc levels were lower in the HD patient compared with controls in a meta-analysis of 128 studies [58]. Moreover, Esmaeili et al. (2019) in a group of 63 children with ESRD on regular HD (78.6 ± 21.6 µg/dL)), 45 on CAPD (74.2 ± 18.1 µg/dL) and 14 in CT (93.5 ± 16.2 µg/dL) highlighted that SZC in the group in HD was significantly lower than in the control group (91 ± 16.4 µg/dL) [59]. Youssef et al. (2012) also revealed that the SZC in children on regular HD was significantly lower than in healthy children or children with CKD in CT [60]. Similarly, Esfahani et al. (2006), pointed out that mean SZC was lower in the group of 40 patients on regular HD than in children on CT and healthy children (*p* > 0.001) [39]. 

Quite the reverse, at the beginning of this study, the mean SZC was in ranges of hypozincemia and lower in CAPD (66.2 ± 18.9 µg/dL) than in HD (76.5 ± 15.1 µg/dL) or CT (71.6 ± 16.2 µg/dL) patients (ANOVA, *p* = 0.429). This situation is also worrying because this specific dialyzed may be the reason why a zinc-deficient status is suffered by these children. Additionally, after ZS, the mean ZSC (73.5 ± 17.4 μg/dL) was normal and corresponded to 24 patients in HD, who were the only ones that completed more than 10 months of ZS. Even though the mean SZC after ZS was slightly lower than at the beginning of the study, there was not a significant difference in neither group A (from 78 to 71.9 μg/dL, *p* = 0.310) nor group B (from 77.9 to 75.4 μg/dL, *p* = 0.505). According to Thompson (1991), this decrease of SZC in both groups, despite ZS may be explained by increased avidity of depleted tissues for zinc, such as bone or muscle, or due to the disease itself [61].

In the NHANES 2011–2014 study, SZC were higher in males (84.9 ± 0.8 µg/dL) than in females (80.6 ± 0.6, *p* < 0.0001) [55]. These facts are in concordance with the results of this study, because at the start and the end of the study, males had a higher mean of SZC (75.6 and 75 μg/dL) than females (74.6 and 72 μg/dL). In addition, in the NHANES 2011–2014 study there was no difference in SZC in those aged between 6 to 9-years-old (81.1 ± 1.1 µg/dL) compared with those aged ≥10-years-old (82.8 ± 0.6 µg/dL, *p* = 0.59) [55]. Similarly, in this study the mean SZC did not show significant differences between patients under 10-years-old (64.7 and 58.8 μg/dL) and ≥10-years-old (75.9 and 74.8 μg/dL). However, despite ZS mean SZC in children under 10 years remained in hypozincemia ranges, which may be because they need a higher dose of ZS. We take into consideration that children are particularly sensitive to ZnD during periods of rapid growth during which a higher need for zinc could exist, which in these children may not be covered [62]. That means that a higher growth rate corresponds to higher net zinc retention [63] because zinc is the most important factor in the needs related to the deposit of new tissue [64].

Henningar et al. (2019), regarding the NHANES 2011–2014 study carried out in the US, concerning the prevalence of low SZC indicated that around 4% and 8% of children and adults, respectively, have low SZC and may be at risk of ZnD [55]. Nonetheless, Roozbeh et al. (2011) pointed out CKD patients are at higher risk for ZnD, with up to 78% of HD patients being deficient [65]. In this study, despite 12-month of ZS, the hypozincemia cases were slightly increased from 40.5% to 41.7%. Likewise, Josey et al. (2018) in a study with 47 children (24 girls) including 19 in CAPD and 28 in HD, median age 11.4 (2.8, 14.4) years, showed that 43% of their patients had a ZnD. They drew attention to the fact that 90% (*n* = 18 out of 20) were receiving zinc or a zinc-containing supplement, without which their zinc levels could have been lower [66].

According to Panel of Zinc Experts WHO/UNICEF/IAEA/IZiNCG (World Health Organization/United Nations International Children’s Emergency Fund/International Atomic Energy Agency/International Zinc Nutrition Consultative Group), if more than 20% of the whole population (or population subgroup) has SZC below the cut-off point for age and sex. This population (or subgroup) should be considered at risk of zinc deficiency and a public health concern and an intervention to improve population zinc status is recommended [18,28,37]. Besides, serum zinc deficiency has been reported in CKD patients due to hypoproteinemia, tubular reabsorption impairment, proteinuria and calcitriol deficiency, which has a role in zinc intestinal absorption [67]. These facts support the idea that CKD children should receive ZS as a part of the treatment protocol.

Zinc is a micronutrient with anti-inflammatory properties [68] and ZnD is associated with a decline in the immune system [69], with increased susceptibility to infections, exaggerated inflammatory responses [68] and inflammation leading to chronicity [69]. Supplementation with zinc has reduced oxidative stress markers, inflammatory cytokines and infection incidence [68]. In a meta-analysis, Mousavi et al. (2018) displayed a significant reduction in circulating CRP levels (*p* ≤ 0.001) after ZS. They concluded that SZ might have a beneficial effect on the serum CRP, especially at a dose of 50 mg/day, and in renal insufficiency adults patients compared with healthy subjects [70]. Nevertheless, in this study, although mean CRP (9.3 ± 7.5 U/L) after ZS was lower than before (22.4 ± 28.1 U/L), there was an increase of high CRP cases (from 40% to 54.5%) after ZS. These differences may be because zinc doses below 50 mg/day were used in this study. 

According to Expert Panel Reviews (2016), in patients with chronic malnutrition and/or acute illness in whom serum albumin is low, this should be considered as another confounding factor to interpret plasma zinc concentration (PZC), because albumin is the primary carrier protein for circulating zinc [18]. Hypoalbuminemia is common in CKD patients associated with an increase in morbidity/mortality in both adults and children [6,71]. In this study, although the mean serum albumin was normal and identical before and after ZS after ZS, there was a slight increase in hypoalbuminemia cases from 36.8% to 37.5%, and two children had hypozincemia and hypoalbuminemia at the same time. This situation is also worrying because Wong et al. (2002) draw attention to the fact that those patients under 18-years-old who start dialysis with hypoalbuminemia are at a higher risk of death. In 1723 children with ESRD identified through the United States Renal Data System, every 1 gr/dL fall of serum albumin at the start of dialysis was associated with a 54% higher risk of death [51,71].

Mean serum albumin was lower in ranges of hypoalbuminemia in patients in CAPD (2.9 ± 1.1 g/dL) than in HD (3.6 ± 0.6 g/dL) and in CT children (3.5 ± 0.8 g/dL; ANOVA, *p* = 0.061). Brem et al. (2002) pointed out that children maintained on CAPD were at greater risk of protein malnutrition compared with peers treated with HD. This may be due in part to losses in peritoneal dialysis fluid [72]. Additionally, the results of this study revealed that there was a positive and significant association between serum zinc and albumin before (*p* = 0.000) and after ZS (*p* = 0.007). By the simple regression analysis, R Square indicates that 41% of the variations of the SZC before (Figure 2) and 32% after ZS (Figure 3) may be explained by the serum albumin. These results are in line with the NHANES 2011–2014 study, in which the SZC had a positive association with serum albumin (*p* < 0.0001) [55]. Moreover, hypozincemia has been reported in patients with CKD owing to hypoproteinemia, proteinuria and tubular reabsorption impairment [67,73,74]. Foote et al. (1984) drew attention to the fact that it was more likely to have SZC below the cutoff for albumin of 3.5 g/dL [75]. Furthermore, serum albumin concentrations responded to zinc supplementation in severely zinc-deficient individuals [76], although this has not been shown in marginally zinc-deficient individuals [77].

In this study, there were no significant differences between mean SZC in patients with high CRP (74.3 µg/dL, *p* = 0.931) and normal CRP in the start (74.9 µg/dL). However, the mean SZC in patients with hypoalbuminemia was significantly lower (67.8 µg/dL, *p* = 0.012) than patients with normal serum albumin (80.6 µg/dL). After ZS, in patients with hypoalbuminemia was even lower (64.3 µg/dL, *p* = 0.072) than in normal patients (78.3 µg/dL). These results are in concordance with NHANES 2011–2014 study, in which patients with hypoalbuminemia (Odds Ratio: 11.2; 99% CI: 3.4, 37.3; *p* < 0.0001) were more likely to have low SZC [55]. Nevertheless, after ZS, mean SZC in patients with high CRP was significantly lower and in ranges of hypozincemia (66.1 µg/dL, *p* = 0.012) than patients with normal CRP (79.9 µg/dL). Corbo et al. (2013) pointed out that acute stress and inflammation may contribute to zinc redistribution, leading to lower PZC [78] without affecting total body store [79]. In a study over 114 patient’s adults with a critical illness, Ghashut et al. (2016) highlighted that PZC was independently associated with both CRP and albumin as markers of the systemic inflammatory response [80].

Results on the effect of two doses of ZS on nutritional status in these CKD children with chronic malnutrition and elevated percentage of hypoalbuminemia, hypozincemia and high CRP cases, indicated that in the whole series, both doses of ZS produced a small but significant increase in body mass, mainly in group A that received 30 mg/day of zinc. In the multiple regression analysis, R Square indicated that 95% of the variation of BMI in group A might be explained mainly by W/A, H/A and FFM. In contrast, the variation of BMI in group B may be explained primarily by W/A. These facts support the idea that contribution of ZS in group A was due to the rise of linear growth, weight and fat-free mass gain. Additionally, there were more patients with positive changes in BMI Z-score and normalization of hypoalbuminemia, hypozincemia and high CRP status in group A than B (Table 6). These outcomes are in agreement with the results of El-Mashad et al. (2019) in 22 children in HD who received 50 mg zinc sulphate twice daily for 90 days, showing improvement in BMI after ZS [81]. Furthermore, El-shazly et al. (2015) observed that body weight and BMI were significantly increased (*p* < 0.001) after ZS [56]. 

The importance of the increase in body mass in this series after ZS is significant since low BMI is associated with increased morbidity and mortality with ESRD [51] and as a predictor of mortality in both adults and children [53]. Nonetheless, in CKD children, the normal relations between age, height and sexual maturation are commonly disturbed, and BMI may not be interpretable in the same way in CKD children as it is in healthy children [82]. Expressing BMI relative to chronologic age results in significant underestimation of relative lean mass (LM) and adiposity in children and adolescents with CKD, and may result in overdiagnosis of underweight [83]. Furthermore, since BMI varies considerably throughout childhood, reaching a trough at 4–6 years of age, it has been suggested that it should be calculated according to height age [52,82]. In the absence of clinically feasible alternatives, BMI-height-age Z-score represents a reasonable tool in the nutritional assessment of children with CKD [83].

Considering this, although the mean BMI-height-age Z-score was normal in all participants before (−0.13 ± 0.42 kg/cm^2^) and after SZ (−0.13 ± 0.4 kg/cm^2^), there was a small and significant change (*p* = 0.033) in all patients and particularly in group A (*p* = 0.022) after ZS. In addition, there was a negative and significant association between BMI-height-age Z-score and serum albumin (*r* = −0.37; *p* = 0.023) before ZS. In this study, to support the effect of ZS on nutritional status, other anthropometric tests were used. The TSF as a measure of subcutaneous fat and MAC as a reflection of muscle mass may be more useful in determining body composition than the calculation of BMI alone. In the whole series, ZS possibly influenced FM (from 7.2 to 8.9, *p* = 0.028) more than MAMA (from 22 to 24.3, *p* = 0.050). However, the FFM was one of the variables that explained BMI after ZS in the multiple regression analysis. This notion is consistent with other reports suggesting that zinc promotes increases in FFM rather than FM [18,84].

In this series, there were no significant differences in FM, FFM by sexes before ZS. However, there was a significant higher FM in females (11 ± 5.4 mm^3^) than males (6.8 ± 3.1 mm^3^, *p* = 0.008) after ZS. In groups of the same chronologic age, Gao et al. (2012) pointed out that when sexually immature CKD females are compared with more developed healthy females, they appear to have inappropriately low adiposity, when in fact their adiposity may be appropriate for their level of maturation. Likewise, CKD males with delayed maturation will appear to have LM deficits compared with healthy boys but with more advanced sexual development [83]. Arsenault et al. (2008) in Peruvian children indicated that the association between zinc status and body composition such as body weight, body fat percentage and lean body mass has been assessed in experimental studies. Similarly, in several studies conducted in children and adolescents, body zinc status was found to affect body composition [85], whereas, in a study of children ages <2-years-old, ZS increased height but had no effect on weight gain [86].

One of the most striking aspects of ZnD is that the available evidence on the impact of preventive ZS on morbidity and mortality in children in lower-income countries, indicate that ZS reduces the incidence of diarrhea by ~20%, acute lower respiratory tract infections (ALRI), reducing pneumonia and ALRI by ~15%. Zinc supplementation may reduce the number of malaria episodes and produces a 6% reduction in child mortality, although this benefit may be restricted to children of 12 months of age or older, in whom the mortality reduction is approximately 18% [87]. Recent meta-analyses revealed that ZS increased linear growth and weight gain in children in developing countries [87,88]. In addition, physical growth argues for the need to develop programs to prevent ZnD in those countries where an elevated risk of ZnD has been identified [87], and it has been previously established that zinc needs to be provided on a daily basis for an extended time [89].

Even though, the literature on pediatric patients with CKD is scarce, we know that ZnD has been reported to be common in these patients and it may be alleviated by ZS with significant health implications [31]. In a meta-analysis of 15 randomized controlled trials, Wang et al. (2017) suggest that ZS benefits the nutritional status of maintenance HD patients and show a time-effect relationship, with an anti-inflammatory effect in these patients [30]. Navarro-Alarcon et al. (2006) highlight that ZS was given to HD patients with low PZC to improve appetite, polyneuropathy, sexual functions, immunological response or even lipid profile [90]. Furthermore, Ohinata et al. (2009) reported that oral ZS improves appetite and stimulates food intake [91]. Similar results were found by Sahin et al. (2009) who stated that zinc supplement to the diets of HD patients may prevent malnutrition [92].

Taking into account the above mentioned, after 12-month of ZS in this randomized study, the real contribution of 30 mg/day of zinc on nutritional status in these CKD children with chronic malnutrition and elevated percentage underweight, stunted growth, hypoalbuminemia, hypozincemia and high CRP cases, was the positive increase of body mass. This increase was seen in the improvement of W/A, H/A, W/H, MAC, NI, BMI, BMI Z-score, BMI-height-age Z-score and FFM. In addition, normalization of more than 25% hypozincemia, hypoalbuminemia and high CRP cases was observed. This dose may be the reason why at least two CKD children, one girl with obesity and another girl with malnutrition and osteodystrophy, improved their nutritional status after ZS. Nonetheless, this dose may not have been enough for other two children, who did not improve their hypozincemia, hypoalbuminemia and high CRP, at the end of the study. Additionally, these results may indicate that children with CKD, hypozincemia and other comorbidities may need doses over 30 mg of zinc per day.

At this point, we must bear in mind that children with CKD in this series have a high risk of continuing with a zinc-deficient state since more than 40% had hypozincemia despite SZ. In addition to the risks inherent in chronic diseases, these children suffer from chronic malnutrition with stunted growth, hypoalbuminemia and inflammation. We must add to this situation the appearance of other comorbidities such as cardiovascular diseases, obesity, diabetes mellitus, dyslipidemias, etc., which may occur in the course of their childhood and adolescence. These health conditions could keep them in a state of high risk of zinc deficiency if proper control and supplementation of this important micronutrient is not carried out.

Therefore, this research is justified and supports the idea that preventive ZS programs in children with CKD should be considered in countries at high risk of ZnD, such as children in the cities of Lima and Callao. Well-designed large multicenter trials are urgently needed to improve understanding of zinc nutritional status in these patients and to determine the adequate amount of zinc. We suggest that pediatric patients with CKD should regularly receive control of their zinc nutritional status and receive at least 30 mg/day of oral zinc as a minimum amount necessary to improve their nutritional status. Additionally, a multicenter international study should be conducted to change the current approach and include the risk of zinc deficiency in primary health prevention. 

The limitations of the present study were that it was partially masked and the sample size, which was reduced to 73% at the end of the study. By design of the study, there was no control group. Furthermore, the second blood sample was obtained from the children in HD who completed more than 10 months with zinc supplements. Although its strengths include the fact that it was a multicenter test study, whose primary and second endpoint were achieved. By design, this study focused on the effect on the administration of two doses of zinc on the nutritional status of children with CKD regardless of the stage of their disease. Fifteen anthropometric and three biochemical evaluations evaluated this nutritional status, and more importantly, a 12-month follow-up of child observation was performed. Doses used in this study were lower than 50 mg/day of zinc (tolerable upper intake level), and it may be the reason why there were no adverse events reported as drug-related in any group of the participants [93].

## 5. Conclusions

Mean serum zinc concentration was normal before and after zinc supplementation. There was a positive and significant association between serum zinc and albumin before and after zinc administration. Zinc supplementation may be beneficial for nutritional status in children and adolescent with CKD due to the fact that participants may have improved their nutritional status through the slight but significant gain in their body mass, especially with 30 mg/day of zinc supplementation.

## Figures and Tables

**Table 1:** The characteristics of kidney disease patients at the start by groups of treatment (day 0, *n* = 48).

Assessments	Group A (*n* = 24)	Group B (*n* = 24)	*p*-Value
Age (years)	13.5 ± 3.2	12 ± 4.7	0.206
Sex (female)	14 (58.3%)	9 (37.5%)	0.155
**Anthropometric**			
Weight-for-age (kg)	32.3 ± 9.9	27.5 ± 12.8	0.146
Height-for-age (cm)	135 ± 18.3	126.5 ± 23.6	0.167
Weight-for-height	33.6 ± 12.6	28.9 ± 9.9	0.189
Body mass index-for-age (kg/cm^2^)	17.4 ± 2.8	16.2 ± 2.7	0.129
Nutritional index	77.2 ± 15.2	73.9 ± 13.6	0.427
Mid-arm circumference (mm)	19.7 ± 3.6	17.9 ± 3.4	0.078
Triceps skinfold thickness (mm)	9 ± 4.7	7.4 ± 2.8	0.156
Mid-arm muscle area (mm^3^)	23.1 ± 8.8	20.9 ± 9.1	0.417
Mid-arm fat mass (mm^3^)	8.4 ± 5.4	6 ± 2.4	0.066
Growth rate (cm/year)	2.6 ± 2.6	3.9 ± 3.4	0.229
Weight-for-age Z-score	−2.3 ± 1.1	−2.6 ± 1	0.270
Height-for-age Z-score	−3.3 ± 1.8	−3.4 ± 1.5	0.761
Weight-for-height Z score	0.8 ± 2.1	−1 ± 2.9	0.062
Body mass index Z-score	−0.8 ± 1.2	−1.2 ± 1.1	0.280
BMI-height-age Z-score	−0.15 ± 0.1	−0.11 ± 0.6	0.744
**Biochemical analysis**			
Serum albumin (g/dL)	3.48 (0.58)	3.58 (0.63)	0.610
C-reactive protein (U/L)	12.2 (20.4)	20 (24.9)	0.356
Serum zinc concentration (µg/dL)	74.9 (14.9)	75.1 (16.5)	0.962
**Comorbidities (%)**			
Undernutrition	8.3% (2/24)	12.5% (3/24)	0.500
Underweight	66.7% (16/24)	87.5% (21/24)	0.084
Stunting	75% (18/24)	91.7% (22/24)	0.122
Wasting	7.2% (1/14)	0% (0/16)	1.000
Obesity	4.2% (1/48)	0% (0/24)	1.000
Hypoalbuminemia	44.4% (8/18)	30% (6/20)	0.279
High C-reactive protein	33.3% (5/15)	46.7% (7/15)	0.355
Hypozincemia	36.8% (7/19)	44.4% (8/18)	0.446

Values are presented as mean ± standard deviation score (SDS).

**Table 2:** Causes of chronic kidney disease in this series (*n* = 48).

Causes	Therapy Zinc Sulphate					
	Group A	%	Group B	%	Total	%
Glomerulonephritis	9	18.75	9	18.75	12	37.50
Obstructive uropathy	6	12.5	7	14.58	13	27.08
Chronic interstitial nephritis	6	12.5	4	8.33	10	20.83
Unknown cause	3	6.25	4	8.33	7	14.58
Total	24	50	24	50	48	100

**Table 3:** Differences between assessments pre and post zinc supplementation in the whole series (*n* = 48).

Assessments	*n*	Pre-Treatment	*n*	Post-Treatment	*p*-Value
**Anthropometric**					
Weight-for-age (kg)	48	29.9 ± 11.6	35	32.6 ± 9.5	0.015 *
Height-for-age (cm)	48	130.8 ± 21.3	35	137.6 ± 14.7	0.000 *
Weight-for-height	42	31.3 ± 11.5	27	34.5 ± 11.1	0.000 *
Mid-arm circumference (cm)	48	18.8 ± 3.6	35	20 ± 3.3	0.035 *
Triceps skinfold thickness (mm)	45	8.2 ± 3.9	35	9.3 ± 3.6	0.093
Body mass index (kg/cm^2^)	47	16.7 ± 2.6	35	16.8 ± 2.5	0.543
Nutritional index	48	75.5 ± 14.3	35	72.53 ± 14.1	0.322
Mid-arm muscle area (mm^3^)	44	22 ± 8.8	35	24.3 ± 8.1	0.050
Mid-arm fat mass (mm^3^)	44	7.2 ± 4.3	35	8.9 ± 4.9	0.028 *
Growth rate (cm/year)	32	3.2 ± 2.9	34	2.3 ± 4.1	0.260
Weight-for-age Z-score	48	−2.43 ± 1.06	35	−2.6 ± 1.1	0.036 *
Height-for-age Z-score	48	−3.34 ± 1.6	35	−3.48 ± 1.72	0.012 *
Weight-for-height Z score	30	−0.15 ± 2.7	20	0.08 ± 1.36	0.469
Body mass index Z-score	48	−1 ± 1.1	35	−1.2 ± 0.9	0.411
BMI-height-age Z-score	35	−0.13 ± 0.4	35	−0.21 ± 0.3	0.323
**Biochemical analysis**					
Serum albumin (g/dL)	23	3.6 ± 0.5	23	3.6 ± 0.5	0.915
C-reactive protein (U/L)	11	22.4 ± 28.1	11	9.3 ± 7.5	0.157
Serum zinc concentration (µg/dL)	37	75 ± 15.5	24	73.5 ± 17.4	0.330
**Comorbidities (*n*, %)**					
Undernutrition	48	5 (10.4)	35	8 (22.9)	0.083
Underweight	48	37 (77.1)	35	28 (80)	0.571
Stunting	48	40 (83.3)	35	29 (82.9)	0.571
Wasting	30	1 (3.33)	20	0 (0)	1.000
Obesity	48	1 (2.1)	34	1 (2.9)	1.000
Hypoalbuminemia	38	14 (36.8)	24	9 (37.5)	0.747
High C-reactive protein	30	12 (40)	11	6 (54.5)	0.588
Hypozincemia	37	15 (40.5)	24	10 (41.7)	0.770

Values are presented as mean ± SDS. (*n*, %) number and percentage of cases. * Significant differences were statistically analyzed by a Student’s *t*-test.

**Table 4:** Statistical changes after zinc supplementation in the whole series (*n* = 35).

2º – 1º Assessments	Z	*p*-Value
**Anthropometric**		
Weight-for-age	−1.02	0.305
Height-for-age	−4.62	0.000 *
Weight-for-height	−4.46	0.000 *
Mid-arm circumference	−0.92	0.355
Triceps skinfold thickness	−1.74	0.080
Body mass index	−2.17	0.030 *
Nutritional index	−1.80	0.071
Mid-arm muscle area	−0.61	0.537
Mid-arm fat mass	−1.46	0.144
Growth rate	−0.11	0.912
Weight-for-age Z-score	−2.10	0.036 *
Height-for-age Z-score	−2.52	0.012 *
Weight-for-height Z score	−0.14	0.891
Body mass index Z-score	−0.82	0.411
BMI-height-age Z-score	−0.33	0.033 *
**Biochemical analysis**		
Serum albumin	−0.00	1.000
C-reactive protein	−1.89	0.059
Serum zinc concentration	−0.97	0.330

* Significant differences were statistically analyzed by a Wilcoxon test.

**Table 5:** Statistical changes by groups of treatment after zinc supplementation (*n* = 35).

Assessments	Group A		Group B	
	Z	*p*-Value	Z	*p*-Value
**Anthropometric**				
Weight-for-age	−1.13	0.257	−0.37	0.705
Height-for-age	−3.18	0.001 *	−3.41	0.001 *
Weight-for-height	−3.06	0.002 *	−3.24	0.001 *
Mid-arm circumference	−2.04	0.041 *	−0.99	0.319
Triceps skinfold thickness	−1.19	0.234	−1.31	0.187
Nutritional index	−2.23	0.025 *	−0.63	0.527
Body mass index	−2.62	0.009 *	−0.43	0.660
Mid-arm muscle area	0.00	1.000	−0.55	0.581
Mid-arm fat mass	0.00	1.000	−1.71	0.086
Growth rate	−0.12	0.905	−0.23	0.811
Weight-for-age Z-score	−2.03	0.043 *	−1.02	0.306
Height-for-age Z-score	−1.48	0.140	−2.16	0.031 *
Weight-for-height Z score	−0.53	0.594	−0.31	0.721
Body mass index Z-score	−0.44	0.660	−1.52	0.129
BMI-height-age Z-score	−1.92	0.022 *	−1.19	0.210
**Biochemical analysis**				
Serum albumin	0.00	1.000	0.00	1.000
C-reactive protein	−1.34	0.180	−1.41	0.157
Serum zinc concentration	−1.16	0.247	−0.16	0.875

Values are presented as mean ± SDS. * Significant differences were statistically analyzed by a Wilcoxon test.

**Table 6:** Differences between percentages of cases with positive or negative change by groups of zinc supplementation (*n* = 35).

Nutritional Assessments	Group A (+)	Group A (−)	Group B (+)	Group B (−)	X^2^ *p*-Value
Body mass index Z-score	16.7 *	5.6	5.8	11.8 *	0.020
Weight-for-age Z-score	12.1	27.8	23.5	29.4	0.243
Height-for-age Z-score	22.2	38.9	17.6	64.7 *	0.074
Hypoalbuminemia	30 *	30 *	8.3	25	0.032
High C-protein reactive	28.6 *	0	9.1	18.2 *	<0.0001
Hypozincemia	30 *	30 *	8.3	25	0.032

Values are presented as percentages. Significant differences were statistically analyzed by an X^2^ test with Yates’s correction and * Fisher’s exact test.
